# Hospital and Institutional News

**Published:** 1920-12-25

**Authors:** 


### HOSPITAL AND INSTITUTIONAL NEWS.

### THE B.M.A. REPRESENTATIVE MEETING.

The financial position of the voluntary hospitals was discussed at length on December 21 at a conference of representatives of the honorary staff of the voluntary hospitals of England and Wales, called by the British Medical Association. Below we summarise the main points of the proceedings. A resolution recording the belief that the voluntary method of administration of the voluntary hospitals was to the advantage of the public, of medical science, and of the medical profession, and that it should be maintained, was carried with only four dissentients. It was also agreed that it is undesirable that the voluntary hospitals should be subsidised by the local rating authorities, except so far as payment is made for the examination and the care of patients for whom these authorities are responsible. After some discussion the motion was also approved that in times of financial difficulty it might be desirable that the Central Government should subsidise voluntary hospitals, and that such subsidies in the case of any individual hospital should take the form of a contribution proportional to the income received by the hospital from voluntary and other contributions, the subsidies being made through some central fund.

### EMPLOYERS AND INSURANCE COMPANIES.

The conference next registered its view that a greatly extended support should be sought from employers and insurance companies, and then an amended resolution was carried to the effect that every in-patient who is not a necessitous person should make a weekly contribution towards the cost of maintenance. Following discussion of some finer points on subscription collection, it was then agreed that it was desirable that the voluntary hospitals, after securing sufficient accommodation for necessitous poor, should make arrangements for the reception of patients who are able to pay in whole or in part. The view was upheld that no fixed rate of payment for professional services rendered to such patients should be established, the fees so payable remaining, as at present, an arrangement between the patient, family physician, and consultant. Finally, it was agreed that the logical position for the members of the honorary staffs of all voluntary hospitals to take up in event of these payment schemes, etc., materialising is that a percentage of all such payments should be passed into a fund which could be allocated in any manner which the honorary medical staff might determine. It was also decided that it should be left to the Hospitals Committee of the British Medical Association to call another conference if this were deemed necessary.

### THE POOR-LAW HOSPITALS.

The main question opened at last week's meeting of the Harveian Society concerned the future of the Poor-law infirmaries. The chief speakers were Dr. Wilson, of St. Mary's Hospital, Dr. Buttar, and Mr. E. W. Morris. Each in his own way indicated the absolute necessity for linking up infirmaries with the big voluntary hospitals, sharing in the main some of the constructive suggestions carried by the much-criticised Dawson Report. No immediate change was, however, anticipated in the voluntary system principle, and it is true that any such departure is at the moment unthinkable to the majority of medical men. Mr. Morris suggested that in future money raised by voluntary subscription should be primarily allocated to research and professional education.

### IMPORTANT MEETING AT BIRMINGHAM.

An important meeting of the Birmingham Chamber of Commerce was lately addressed by Sir David Brooks and Sir Gilbert Barling on behalf of the Hospital Council. Mr. H. C. Field presided. It was finally resolved to urge all citizens to support the voluntary hospitals, to recommend the General Purposes Committee of the Chamber of Commerce to consider how best to bring the needs of the hospitals to the notice of their members, while the resolution declared against the assumption of hospitals by the State and municipality. Mr. Field began by stating that the Chamber had always been opposed to official control of any sort, and consequently the object of the meeting would be endorsed. The present deficit on the Birmingham hospitals was £65,000, Sir David Brooks remarked. Since the war the contributions of the Hospital Saturday Fund had doubled; other subscriptions had shown only a small improvement. An employer should not only encourage his staff to subscribe, he should set an example. His own suggestion was that employers should give out of their gross profits a sum equal to the amount contributed by their workpeople. He said "gross profits" because these contributions would benefit the workpeople and would be reckoned in trading expenses before liability to taxation was assessed. The Hospital Council had formed a guild to gain help from shops, offices, banks, insurance companies, and all concerns not already subscribing through the Hospital Saturday Fund.

### SIR GILBERT BARLING'S PLAN.

Sir Gilbert Barling emphasised the present predicament by remarking that hospitals were being compelled to sell their investments. He hoped still that the £60,000 required would be forthcoming. The income of the Sunday Fund, already improved by the new organisation, ought to reach £10,000, or twice the present total, when the new machinery was perfected. Of the Saturday Fund, Sir David Brooks had spoken already. The insurance companies owed much of the profits to the fact that their policyholders, when victims of accident or illness, were treated at the voluntary hospitals. He thought also that unmarried patients, and others who could afford to pay, should help with 10s. or £1 a week during their stay. A third source of help existed. In Birmingham the number of State-insured persons was between 300,000 and 400,000, or 13,000,000 in the whole country. The contributions taken by the State from employees and workpeople were largely paid to panel doctors. But the hospitals provided the most important part of the treatment for nothing. If the State fulfilled its bargain it should give to the voluntary hospitals something for the work which they did. If the State gave a capitation fee of 1s. for every insured person, the contribution in Birmingham alone would amount to £15,000. If his suggestion of a capitation fee were allowed, the income, with other sources, would keep the hospitals going.

### THE LEAGUE OF MERCY.

The twenty-first annual meeting of Presidents of the League of Mercy was held at St. James's Palace on Monday, December 20, and was presided over in the absence of H.R.H. the Prince of Wales by the Lady Grand President, H.R.H. Princess Alice, Countess of Athlone. H.R.H. was supported by H.H. Princess Helena Victoria and H.H. Princess Marie Louise, while among those present were the Dowager Lady Dimsdale, Sir Wm. and Lady Collins, Lord Farquhar, Lady Alexander, and Lady Tree. The minutes of the last meeting having been read and approved, Mr. Montagu Sharpe, K.C., D.L., moved that the Rt. Hon. the Viscount Farquhar, G.C.V.O., be appointed Chairman, Sir Frederick Green, K.B.E., Deputy-Chairman, and Mr. Arthur H. Greening Hon. Secretary respectively. After this motion had been carried, H.H. Princess Marie Louise, Col. the Rt. Hon. Lord Lambourne, the Dowager Lady Dimsdale, O.B.E., Viscount Hill, Lady George Lawson-Johnston, and the Rev. H. Hornby-Steer, M.A., were appointed members of the Executive Council. H.R.H. the Grand President then presented the Bar of the Order of Mercy and the Order of the League to a number of ladies and gentlemen, and in delivering the Presidential address, read letters from H.M. the King and H.R.H. the Prince of Wales. Sir Frederick Green, in reviewing the work of the League during the year, explained that the total has reached £25,483, and that yet greater success may be achieved in the future. (See also p. 295.) Of the very excellent speeches and the presentations full details will be published in our next number.

### A RECORD COLLECTION.

The annual meeting of the constituents of the Metropolitan Hospital Sunday Fund, held at the Mansion House on December 17 under the presidency of the Lord Mayor, claimed a large attendance. According to the annual report the year's collection has resulted in a total of £121,058, the largest figure ever attained and exceeding that of the previous twelve months by £34,826. At the various places of worship a sum of £53,486 was collected, and donations, including £5,045 collected at Lloyd's and £4,000 from the Rt. Hon. the Lady Strathcona and Mount Royal, amounted to £31,006. Working expenses for 1920 were slightly increased; £3,828 19s. 9d. against £3,398 16s. 11d. for 1919, but they will be found to form only 3.162 per cent. of the gross receipts, as compared with 3.491 per cent. last year. It is to be noted that while the expenses due to printing, postage, &c., have greatly increased, the percentage cost of the collection and distribution of the fund in 1920 is lower than it has ever been. As was remarked by Mr. R. Holland-Martin, the vice-president of the fund, and to whom much credit for its prosperity is due, this record collection is only a part of what may be achieved in the future. Great help has been derived from the keen interest taken by the late Lord Mayor, Sir Edward Cooper, in the fund, and it is hoped that the collections for the coming year may reach at least £150,000. Of the sum raised in 1920, already £110,000 has been paid to the various hospitals. The great success of house to house collections was commented upon by the Rev. G. C. Wilton, who urged that local committees, representing all denominations, should be appointed to carry on this excellent work. In explaining the great future possibilities, the Rev. Thos. Jackson remarked that it was a noteworthy fact that a poor slum mission in the East End had done so remarkably well for the fund that it was twelfth in the list for the whole of London. Following the reappointment of the Council, it was decided, on the motion of the Archdeacon of London, that Hospital Sunday 1921 should be held on June 26.

### THREE IMPORTANT HOSPITAL APPOINTMENTS.

The Cancer Hospital has made public the fact that both the office of Director of the Research Institute and that of Director of the Pathological Department will fall vacant on March 1, 1921, and invites applications. The salary of the Director of Research is £1,200 a year, and £800 is offered in respect of the other post. Following upon the lamented death of Mr. Guy Dale, the vacant post of Secretary to the Metropolitan Hospital is advertised, and candidature is restricted to men with hospital experience. The salary offered, £550, is rather on the modest side, and will hardly attract anyone of much standing in the hospital world. As may be expected, the new officer is needed at once, this and the coming month being busy times on the administrative side.

### WHEREFORE TARRY YE?

As announced in our issue of December 4, the Minister of Health has promised to appoint an independent and impartial committee of inquiry of five suitable people who are not medical practitioners or otherwise identified with hospital management to inquire into the exact economic position of voluntary hospitals at the present moment. As none of the interests of professions, societies, and institutions, such as are so troublesome to adjust in many cases of choosing members of inquiry committees, are involved, we do not quite see why there should be so much delay in dealing with a matter which is admitted on all sides to be urgent.

### THE LATE MR. J. S. WOOD.

On December 20 Mr. Joseph Snell Wood died at Kensington Court after a long illness. Mr. Wood was a keen supporter of the voluntary system who never wearied in well-doing, and is reputed to have raised by various schemes which he initiated or organised not less than half a million for hospital charities. He had been on the Council of the Chelsea Hospital for Women for thirty-four years, was for five years honorary secretary of the Bolingbroke Hospital, Wandsworth, and had also been a member of the Council of the British Hospitals Association. Nor were these all his activities in the hospital world: he founded the Children's Salon, and was also connected with King Edward VII. Hospital Fund for London. Mr. Wood was a journalist by profession, and took a keen interest in the well-being of that craft; he was also chairman and managing director of the *Gentlewoman Illustrated*. He was in his sixty-eighth year at the time of his death.

### THE R.A.S.C. MEMORIAL FUND.

THE General Committee of the Royal Army Service Corps Memorial Fund are prepared to pay the cost, or part of the cost, of convalescent treatment for men who have served in the R.A.S.C. who are medically recommended for convalescent treatment and are unable to afford such treatment for themselves. The General Committee would be grateful if secretaries of hospitals would bring such cases to the notice of the hon. secretary of the fund, Kensington Palace Barracks, W. 8.

### THE BIRTHDAY LEAGUE IN SOUTH LONDON.

MR. R. J. COLES, Superintendent of King's College Hospital, has lately been fertile in suggestions for raising funds for London hospitals. His latest plan, for the benefit of King's College Hospital, is to start a Birthday League for all wage-earners in South London. The object is for each member to promise a gift of four shillings a year upon his birthday, a sum which nearly corresponds to a penny a week contribution. The appeal secretary of the hospital is in charge of the scheme, and post-cards with designation, name, and address should be sent to him at the hospital. The objection that these schemes tend to overlapping is answered by the observation that while one form of a scheme often falls flat, another form of the same idea becomes popular, and each hospital at the present time must discover by experience which form of contribution best suits the district in which it is situated.

### A HOSPITAL STORY.

THE enlarged monthly *Journal* of the Metropolitan Hospital Saturday Fund has produced a Christmas Number, in which we are glad to see Mr. E. W. Morris as an author. His short story, "A Queen in Disguise," is that unusual thing, a good hospital story which steers between the rocks of sentiment and unreality and yet gives to the hospital a warm place in the reader's mind. The story is founded on a mistake, a serious mistake, which led to an inquest, and this touch of reality, since mistakes will happen in spite of every care, at once holds the attention of the reader. We wish more of this kind of writing were obtainable, for, in truth, most hospital stories are poor stuff. If the Saturday Fund *Journal* can bring more such contributors to light it will deserve our gratitude, for of the mass of incident that occurs in hospital life, how little gets into print, and how rare is the pen which knows how to treat it properly! Mr. Morris is believed to be a very busy man, but it is to be noted that he finds time to write short stories, just as he found time in the past to write a history of the London Hospital.

### THE RADCLIFFE INFIRMARY.

THE following appointments have been made at the Radcliffe Infirmary, Oxford: (1) As consulting physician, Sir Archibald Garrod, C.M.G., D.M., F.R.C.P., Regius Professor of Medicine, Oxford; (2) as assistant obstetric physician, William Duncan Sturrock, Esq., D.M.; (3) as administrator, A. G. E. Sanctuary, Esq., M.A., steward to the "Dreadnought" Hospital, Greenwich. Mr. Sanctuary was educated at Sherborne School and Gonville and Caius College, Cambridge. He took a commission in the R.F.A. in September 1914, served in India with the 1/3 Wessex Brigade, in Mesopotamia with the 50th Brigade, in Egypt, and France. He was promoted captain in 1917 and disembodied in July 1919. He has held the post of steward at the Seamen's Hospitals, Greenwich, since 1919, where he has controlled the lay staff, arranged the supply of food and other necessaries for 415 beds, and taken part in the general work of the secretary's office.

### BETTER NEWS IN EDINBURGH.

SPEAKING at the second annual meeting of the League of Subscribers to the Edinburgh Royal Infirmary, Mr. Sheriff Creole, Chairman of the board of management, congratulated the League on having raised £13,000 for the Royal Infirmary. The workers also have subscribed over £30,000, and though the cost of the infirmary this year will amount to £130,000, this enormous sum should be very nearly met by the subscriptions received. So satisfactory a result has not been reached for many years, and the infirmary has also received £60,000 from legacies over £100. Mr. Creole says with confidence that this year the voluntary system has triumphed. Speaking for himself, he is opposed to charges for maintenance. The time to ask for help is when a man is well. He hopes the hospital will continue on voluntary lines; and the success of the League after two years shows the vitality of the voluntary principle.

### THE NEW SITE OF DONCASTER INFIRMARY.

THE Committee of the Doncaster Infirmary has for long been considering the question of the new site for the hospital. At a meeting of governors which was well attended, it has been decided to adopt the recommendation of the committee in favour of the hall and grounds left by the late Miss Beckett Denison for the new infirmary. Since, however, the testator placed the governors under no obligation to adopt this site, but empowered them to deal with the property in the best interests of the infirmary, other sites were also considered, such as the Bessecar site of ten acres three miles south of the town, which was offered for £3,000. Mr. W. Clark proposed the purchase of this site at the recent meeting, but the governors decided against him, and the Beckett Denison site was eventually approved by a large majority.

### MEDICAL FEES FOR POOR-LAW PATIENTS.

A difficulty has arisen between the Newton Abbot Hospital and the Newton Abbot Board of Guardians, because the hospital has declined to admit patients from the Ashburton district, which is not within the hospital's area, unless a fee for operations is paid. The Guardians say that, in this case, they will discontinue their subscription, unless such cases are admitted in the event of another hospital being closed. To this the hospital replies that its refusal is conditioned by its rules which are applied to the area granted by private subscribers. Thus to comply with the guardian's request would mean, says the hospital, loss of control over the admission of patients. Beside this, the medical officers are stated to consider that their services should not be given without fee to persons maintained by public funds. It would seem that the difficulty has arisen because the Ashburton area is not served by its own local institution. The question then is: Must a hospital in return for a guardians' subscription receive patients without charge from any district from which the guardians care to send them? If it is only because of a temporary difficulty that Ashburton patients are sent to Newton Abbot Hospital, then perhaps the hospital can stretch a point. But on the other hand a possibly inadequate subscription from the guardians cannot fairly be held to allow them to send patients from anywhere. The matter as we understand it, from a not very clear report, will only be prejudiced if the doctors' desire for fees in certain cases is made the crux of the controversy. Why is Ashburton now without hospital accommodation for its Poor-law cases?

### THE AMERICAN PLAN.

After a recent visit to America, Dr. T. Watts Eden has come back convinced of the advantages of the hospital system of that country. In a public statement he has urged the advantages of classified wards for paying patients, and of the system whereby the American trade unions make annual contributions to cover the cost of treating their members, and the local authority subscribes in order to reserve beds for those unable to pay anything for themselves. We have often commented on the advantages of the American plan, and urged that development should take a similar line in this country. But such a change can only be gradual, because of the present shortage of beds and the lack of co-ordination between the voluntary and municipal branches of our hospital system. It is too much to declare in favour of the American plan, and to describe it, as Dr. Eden seems to do, as an available alternative. Our pay wards will grow in number, but they are only one side of a development which must be general.

### DORSET HOSPITAL ASSOCIATION.

Dorsetshire has done well to take the lead by forming an Association of Voluntary Hospitals for the county. The Dorset Association, formed at a meeting over which Lord Ellenborough presided, will comprise the Dorset County Hospital; the Royal and Princess Christian Hospitals, Weymouth; the Royal Eye Infirmary, Weymouth; the Cornelia Hospital, Poole; the Bridport Hospital; the Swanage Cottage Hospital; the Blandford Cottage Hospital; the Yeatman Hospital, Sherborne; and the Wimborne Victoria Hospital. With Lord Shaftesbury, the Lord-Lieutenant, as President, and Mr. Philip Belben, chairman of the Cornelia Hospital, Poole, as honorary secretary, the Association should accomplish much useful work.

### DARTMOUTH COTTAGE HOSPITAL.

An attempt is being made to reopen the Dartmouth Cottage Hospital, which has been closed through want of funds and some difficulty with the staff. A committee of inquiry, appointed to prepare a scheme for reopening the hospital, has reported that £1,000 a year is necessary to run the hospital, of which £120 is provided by endowment, and that the trade unions have offered to subscribe 2d. per week for each adult member and 1d. per week for each apprentice in return for free treatment for themselves and their families. The Committee recommended that each subscriber of half a guinea be treated on the same terms, and that naval and mercantile marine patients be charged 10s. a day (exclusive of medical fees); and all others to be charged by a scale fixed by the House Committee. A general committee of twenty men and twenty women, with a trade-union representative for every £25 subscribed, would appoint a visiting and a finance committee of four members each; and that these two with the rota doctor for the week should form the House Committee. The report was referred back till the financial support is known in detail. It seems, however, that certain jealousies concerning the old Committee still exist; but we hope that these will not prevent the hospital from being reopened on a proper basis.

### THIS WEEK'S DRUG MARKET.

Business is practically at a standstill as is usual during Christmas week, but this year it is not just a temporary suspension of business but an accentuation of a quiet state of affairs, and seems to have become almost chronic. A considerable number of drugs have again depreciated in price, but price reductions do not seem to tempt buyers. Among the articles that are lower are star aniseed oil, cod-liver oil, caffeine, sandalwood oil, clove oil, permanganate of potash, phenacetin, cocaine, coca butter, and guaiacol carbonate. The price of chloroform has also been reduced. German competition in certain medicinal products is becoming more noticeable, and this accounts to some extent for price depression in various directions.

